# The Bidirectional Relationship Between Type 1 Diabetes Mellitus and Obesity in Pediatric Patients: A Systematic Review

**DOI:** 10.3390/children13060744

**Published:** 2026-05-27

**Authors:** Cătălina Mărgineanu, Lia-Oxana Usatiuc, Maria Lucia Sur, Mara Similie, Alexandru Cristian Bolunduț, Csilla-Enikő Szabo, Dana-Teodora Anton-Păduraru, Gabriela Roman

**Affiliations:** 1 Department of Mother and Child, Pediatrics 1, “Iuliu Hațieganu” University of Medicine and Pharmacy, 400006 Cluj-Napoca, Romania; catalina.colt@elearn.umfcluj.ro (C.M.); sur.maria@umfcluj.ro (M.L.S.); alexandru.bolundut@umfcluj.ro (A.C.B.); csilla.szabo@umfcluj.ro (C.-E.S.); 2 First Pediatric Clinic, Emergency Childrens Hospital, 400370 Cluj-Napoca, Romania; mara.similie@elearn.umfcluj.ro; 3 Pathophysiology, Department of Functional sciences, Faculty of Medicine, University of Medicine and Pharmacy “Iuliu Hațieganu”, 400006 Cluj-Napoca, Romania; 4 Faculty of Medicine, “Grigore T. Popa” University of Medicine and Pharmacy, 700115 Iasi, Romania; dana.anton@umfiasi.ro; 5 Third Pediatric Department, St. Mary Children’s Emergency Hospital, 700309 Iasi, Romania; 6 Department of Diabetes, Nutrition and Metabolic Diseases, “Iuliu Haṭieganu” University of Medicine and Pharmacy, 400006 Cluj-Napoca, Romania; groman@umfcluj.ro

**Keywords:** type 1 diabetes mellitus, obesity, double diabetes, pediatric, review

## Abstract

**Highlights:**

**What are the main findings?**

- Obesity and insulin resistance may accelerate the progression and earlier clinical manifestation of T1DM in genetically susceptible children.
- Overweight and obesity are highly prevalent after T1DM diagnosis and are associated with increased cardiometabolic risk and the emergence of a double diabetes phenotype.

**What are the implications of the main findings?**

- Weight management and cardiometabolic risk reduction should be integrated into routine pediatric T1DM care alongside glycemic control.
- Future prospective studies are needed to clarify causal mechanisms and develop targeted prevention and treatment strategies for children with T1DM and obesity.

**Abstract:**

**Background/Objectives:** The rising prevalence of childhood obesity has coincided with increasing incidence of type 1 diabetes mellitus (T1DM), raising questions regarding their potential bidirectional interaction. This systematic review evaluated the association between obesity and T1DM risk, as well as post-diagnostic weight trajectories and metabolic outcomes in pediatric populations. **Methods:** A systematic review was conducted in accordance with PRISMA 2020 guidelines. PubMed, Embase, and Scopus were searched for studies published between January 2010 and January 2026. Eligible studies included observational and interventional research in children and adolescents addressing T1DM and obesity; reviews, case reports, and non-English studies were excluded. Risk of bias was assessed using Joanna Briggs Institute tools. Due to heterogeneity, results were synthesized narratively. **Results:** Sixty-seven studies were included. Population-based data showed a positive association between higher BMI and incident T1DM, with obesity associated with a twofold increased risk (HR 2.05, 95% CI 1.58–2.66) and a 25% increase per 1-SD BMI increment. Insulin resistance (IR) indices correlated with BMI and predicted faster progression to clinical T1DM in autoantibody-positive individuals. At diagnosis, 20–30% of children were overweight or obese, increasing to 30–40% during follow-up. Excess adiposity was associated with higher insulin requirements and increased prevalence of hypertension and dyslipidemia. Longitudinal data indicate that BMI standard deviation scores rise with age, diabetes duration, and pubertal stage, with higher insulin doses and intensive insulin therapy contributing to weight gain. Conversely, some large cohort studies report no linear association between BMI and incident T1DM, indicating heterogeneity across populations. The limitations of this review include the predominance of observational studies, heterogeneous methodologies, and limited generalizability beyond predominantly European and North American pediatric populations. **Conclusions:** Overall, the evidence supports a bidirectional relationship: obesity may increase T1DM risk and accelerate disease progression, while T1DM-related factors promote weight gain after diagnosis. These findings highlight the importance of integrating weight management strategies into routine pediatric T1DM care.

## 1. Introduction

Type 1 diabetes mellitus (T1DM) is a chronic metabolic disorder characterized by the loss of pancreatic β-cell mass, leading to insulin deficiency and persistent hyperglycemia. Historically considered a condition primarily affecting lean individuals, recent trends have revealed a growing overlap between T1DM and excess body weight in pediatric patients [1,2]. Several epidemiological studies are reporting a rising prevalence of overweight and obesity among children and adolescents with T1DM, signaling a notable shift in disease phenotype [3]. Although the coexistence of obesity and T1DM is increasingly recognized, there is limited synthesis of evidence regarding the mechanisms underlying this bidirectional relationship, particularly in pediatric populations.

In children diagnosed with T1DM, the interplay with obesity appears to be complex and multifactorial. On one hand, intensive insulin therapy—essential for glycemic control—can promote anabolic processes and weight gain, particularly when exogenous insulin doses exceed metabolic demands [2]. This may contribute to fat accumulation and an elevated body mass index (BMI), even in the absence of excessive caloric intake. On the other hand, obesity itself may play a pathogenic role in the development and progression of autoimmune diabetes [1]. Adipose tissue is a metabolically active organ that secretes pro-inflammatory cytokines and adipokines, such as leptin and resistin, which can enhance systemic inflammation and potentially influence immune-mediated β-cell destruction in genetically predisposed individuals [4].

Moreover, children with T1DM often exhibit dietary patterns and lifestyle behaviors that may increase the risk of weight gain. These include disordered eating behaviors, frequent meal skipping, high fat intake, and reduced levels of physical activity—all of which have been independently associated with increased adiposity in this population. Sleep disturbances, another commonly overlooked factor, may further exacerbate metabolic dysregulation [5].

The implications of this dual burden are clinically significant. Excess adiposity in children with T1DM has been linked to poor glycemic control, IR (IR), hypertension, dyslipidemia, and early signs of atherosclerosis [5]. These complications may accelerate the progression of cardiovascular disease (CVD), further compounding the long-term health burden of individuals affected by T1DM. In turn, the presence of obesity may mask classical features of T1DM, delay diagnosis, and impact clinical decisions around insulin dosing and monitoring [6].

The “double diabetes” phenotype, where autoimmune β-cell failure coexists with features of IR, is increasingly recognized as a unique and challenging clinical entity [7]. Understanding the mechanisms, prevalence, and outcomes associated with this overlap is essential for improving prevention strategies, therapeutic approaches, and long-term outcomes [2].

This systematic review aims to evaluate the nature of the bidirectional relationship between T1DM and obesity in pediatric populations by summarizing current findings on their epidemiological association, shared risk factors, and potential pathophysiological mechanisms. The findings will help inform future research directions and guide clinical practices aimed at optimizing care for this vulnerable group.

## 2. Materials and Methods

### 2.1. Search Strategy and Study Selection

This systematic review was conducted in accordance with the PRISMA (Preferred Reporting Items for Systematic Reviews and Meta-Analyses) guidelines. Literature searches were performed on three of the major databases: PubMed, Embase, and Scopus. The search included studies published between January 2010 until January 2026. The search was performed according to the Boolean information retrieval method. The following key terms were used: “type 1 diabetes mellitus” OR “pediatric diabetes” AND “obesity” OR “overweight” AND “pediatric” OR “paediatric” OR “children” OR “adolescent” OR “teen”. The title and abstract of all the studies were screened for relevancy. We also searched the references of all included studies to find additional studies that could be incorporated into our review. Duplicates were excluded using a bibliographic citation management software (EndNote 20, Thomson Reuters, New York, NY, USA).

### 2.2. Eligibility Criteria

To ensure the relevance and methodological rigor of the included studies, predefined inclusion and exclusion criteria were applied. Studies were eligible if they investigated pediatric or adolescent populations with T1DM and included data on overweight or obesity, related metabolic outcomes, or associated risk factors. Only original research articles with observational aspects (cohort, case–control, or cross-sectional) were considered. Randomized controlled trials were included only when they provided relevant epidemiological or descriptive data, as treatment effects were not the primary focus of this review.

Studies were excluded if they did not specifically address T1DM populations, focused exclusively on type 2 diabetes mellitus (T2DM), adult populations, or did not report outcomes related to body weight, adiposity, or metabolic characteristics. To enhance comparability and reduce heterogeneity, the review was restricted to studies conducted in North America and Europe, where healthcare systems, diagnostic criteria, and epidemiological surveillance are relatively comparable. This restriction also reflects the predominance of Caucasian populations in these regions, allowing for more consistent interpretation of findings. Studies from other regions were excluded due to potential differences in genetic background, healthcare access, and disease reporting.

Only articles published in English were considered. Additionally, studies without accessible full texts or unavailable through institutional or interlibrary services were excluded. Conference abstracts, editorials, commentaries, and case reports were also excluded to ensure inclusion of high-quality evidence.

### 2.3. Risk of Bias and Quality Assessment Evaluation

The risk of bias assessment was conducted independently by two reviewers, with disagreements resolved through discussion and verification by a third reviewer. The methodological quality of the included studies was evaluated using the Joanna Briggs Institute (JBI) critical appraisal tools, selecting the checklist appropriate to each study design (cross-sectional, cohort, case–control, or interventional). The JBI tools assess key domains including the clarity of inclusion criteria, validity and reliability of exposure and outcome measurement, identification and management of confounding factors, adequacy of follow-up, and appropriateness of statistical analysis. Based on the overall pattern of responses across checklist items, studies were categorized as having low, moderate, or high risk of bias.

### 2.4. Data Analysis

A meta-analysis was considered inappropriate due to the substantial heterogeneity across the included studies in terms of exposures, methodological approaches, outcome variables, and reported effect measures. Consequently, a narrative synthesis was developed, structured according to the types of exposures examined.

## 3. Results

### 3.1. Selection and Characteristics of the Study

**Figure 1:**
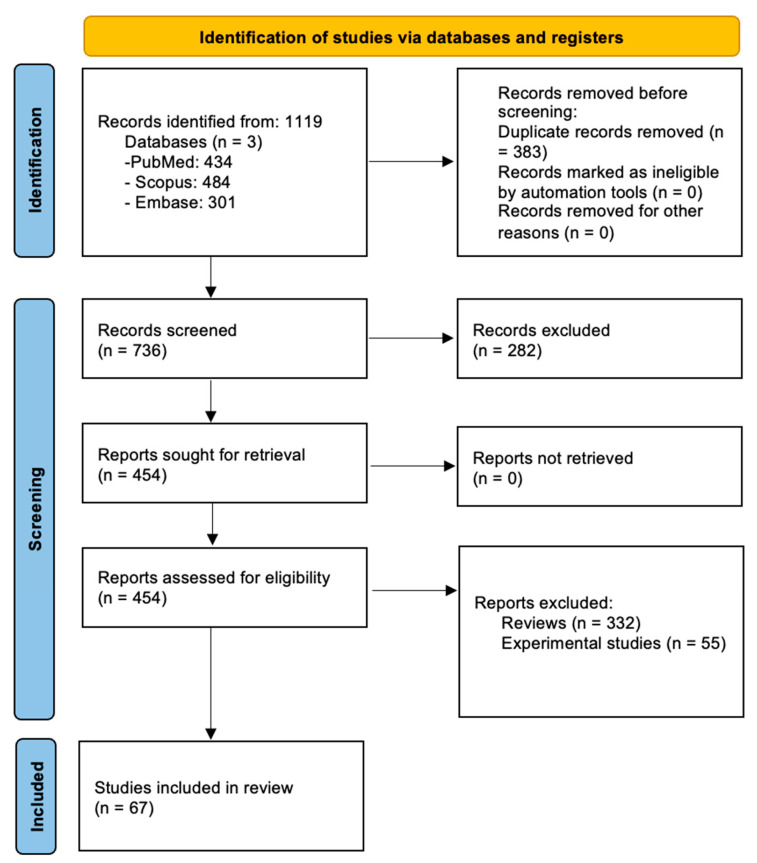
PRISMA Flow-Diagram.

The PRISMA (Preferred Reporting Items for Systematic Reviews and Meta-Analyses) flow diagram (Figure 1) outlines the structured and methodical approach used to identify and select studies for inclusion. An initial total of 1109 records were retrieved from electronic databases—PubMed (n = 434), Scopus (n = 301), and Embase (n = 384). No additional sources were used beyond these databases. After the removal of 373 duplicate entries, 736 unique records were screened. The primary reason for exclusion at this stage was lack of relevance to the review topic (n = 282). Further exclusions included systematic reviews (n = 332) and animal studies (n = 55), in order to maintain a focus on original human research. In the end, 67 studies fulfilled all predefined inclusion criteria and were selected for the final synthesis, thereby contributing to the methodological rigor and reliability of this review.

### 3.2. Quality Assessment and Risk of Bias of the Included Articles

Across the 67 included studies, the overall risk of bias was moderate, reflecting the predominance of observational study designs. Of these, 49 were cohort studies, 11 were case–control studies, and 5 were randomized controlled trials. Most studies met JBI criteria for clearly defined inclusion criteria, objective measurement of exposure (BMI, BMI z-score, or BMI-SDS), and appropriate statistical analysis. However, several domains showed recurrent limitations.

Among cross-sectional and registry-based studies, the most common source of bias was related to temporality, as these designs do not permit determination of whether obesity preceded associated metabolic or clinical outcomes. In addition, outcome assessment for cardiometabolic conditions was often based on routinely collected clinical data, which may vary by site and screening practices. Incomplete adjustment for potential confounders, including pubertal status, insulin regimen, and socioeconomic factors, was frequently observed.

Cohort and longitudinal studies generally demonstrated stronger methodological quality but were affected by incomplete reporting of follow-up or attrition in some cases. Although confounders were commonly identified, analytical strategies to address them were inconsistently described. Missing data handling was variably reported.

Interventional studies exhibited lower risk of selection bias but were limited by small sample sizes and lack of blinding. These factors contributed to a moderate overall risk of bias despite standardized outcome measurement.

Overall, the JBI appraisal indicated moderate risk of bias across studies, with limitations primarily related to study design, confounding, and reporting of follow-up.

### 3.3. Findings of Obesity as a Risk Factor for Type 1 Diabetes Mellitus

The evidence establishing obesity as a causative factor for T1DM centers on the “accelerator hypothesis,” which proposes that weight-related IR increases the metabolic demand on pancreatic beta-cells [8,9]. This overwork triggers a cascade of glucotoxicity and lipotoxicity, stressing the cells and increasing their immunogenicity, which accelerates autoimmune-mediated destruction in genetically predisposed individuals. In the era of full-scale genomic studies, Mendelian randomization has provided causal evidence for this link, showing that a genetically predicted 1-standard-deviation increase in childhood BMI is associated with a 32% higher risk of developing T1DM (OR = 1.32). Epidemiological data from a cohort of 1.4 million adolescents further supports a graded relationship, where obesity doubles the risk of incident disease (HR = 2.05) compared to those with a normal BMI [9]. Furthermore, metabolic markers like higher HOMA-IR (HR = 1.98) and lower Matsuda Index (HR = 0.46) act as “tempo regulators,” where increased IR significantly shortens the transition from pre-symptomatic autoimmunity to overt Stage 3 T1DM [10]. This causative pathway is reinforced by the inflammatory nature of adipose tissue, which secretes pro-inflammatory cytokines that enhance systemic inflammation and facilitate the loss of beta-cell mass.

Across the studies examined, obesity emerges as both a potential risk factor for the development of T1DM and a significant modifier of its clinical trajectory. Evidence is drawn from large population-based cohorts, autoantibody-positive individuals at increased genetic risk, and pediatric cohorts followed longitudinally from diagnosis into adolescence [11,12].

Longitudinal data in autoantibody-positive relatives provide mechanistic support that obesity-related IR accelerates progression through pre-symptomatic stages of T1DM. In the TrialNet Pathway to Prevention cohort, Petrelli et al. studied 6256 first- and second-degree relatives of people with T1DM who were islet autoantibody-positive at baseline. HOMA-IR (IR) and the Matsuda Index (insulin sensitivity) were strongly associated with both age and BMI percentile. After adjusting for insulin secretion (Index 60 or insulinogenic index), higher HOMA-IR and lower Matsuda Index significantly increased the risk of transition from Stage 1 (≥2 autoantibodies with normoglycaemia) to Stage 2 (dysglycaemia) and from Stages 1/2 to Stage 3 (clinical diabetes). For example, for progression from Stage 1 or 2 to Stage 3, each tertile increase in HOMA-IR yielded an HR of 1.98, whereas higher Matsuda Index was protective (HR 0.46) [11].

Several pediatric cohorts describe the weight status and metabolic phenotype at or shortly after diagnosis, providing indirect insight into obesity as a risk factor. A Spanish cohort found that children with T1DM and excess body mass tended to be diagnosed at a slightly younger age than their normal-weight peers, although BMI-Z at diagnosis was not correlated with age overall [13].

Multiple studies from Europe and North America report that 20–30% of children present overweight or obese at diagnosis, despite the catabolic state that typically precedes recognition of T1DM, suggesting that many were already overweight before symptom onset [14,15,16].

At the same time, In a large UK population-based cohort including 369,362 children and young adults, the incidence of both T1DM and T2DM increased over the study period; however, rising obesity was strongly associated with incident T2DM but not with T1DM [17]. While obese individuals had a markedly higher risk of developing T2DM, no positive linear association was observed between BMI and incident T1DM.

From this perspective, the global childhood obesity epidemic has been viewed as a potential driver of earlier onset T1DM.

### 3.4. Post-Diagnostic Weight Trajectories and Prevalence of Obesity in Youth with Type 1 Diabetes Mellitus

Evidence from large registries and longitudinal cohorts consistently shows that overweight and obesity are common among children and adolescents with T1DM and frequently increase after diagnosis [18,19,20]. Across European and North American cohorts, the combined prevalence of overweight and obesity in youth with T1DM typically ranges from 30% to 40%, with obesity alone affecting 10–20% of patients, depending on age, sex, and country [19,21,22,23,24,25,26]. These proportions are comparable to, and in some settings higher than, those observed in the general pediatric population (Table 1).

In a Belgian cohort of 390 children followed from diagnosis (1991–2015), De Keukelaere et al. observed a significant rise in BMI standard deviation score (BMI-SDS) as a function of both age and time since diagnosis, with stronger associations in girls. Children diagnosed after puberty had the steepest BMI-SDS increase [8,27].

An international study from Australia, North America and Europe including 11,513 youth with T1DM identified multiple BMI Z-score trajectories, with a sizeable subgroup showing persistently high or progressively increasing BMI throughout childhood and adolescence [28].

In a retrospective review of American youth, the rate of overweight/obesity increased from 35.5% at diagnosis to 52.8% five years later (Table 2). Research identifies puberty as a particularly high-risk period for weight gain, with the average age for rising into the overweight or obese category being 12.7 years. This trajectory is often accompanied by an increase in mean HbA1c (from 8% to 8.4%) and higher insulin requirements, signaling the development of IR [29].

Case–control data from northeast Poland (85 children with T1DM and 84 controls) showed that median BMI was higher in the T1DM group (19.2 vs. 17.8 kg/m^2^, *p* < 0.05), and abdominal obesity (waist circumference ≥ 90th percentile) was more frequent in T1DM (27% vs. 12%) [30].

In a nationwide Danish registry study including 6097 children with T1DM, median BMI z-scores were elevated in both girls (0.85) and boys (0.67) over the study period. A non-linear association was observed between BMI z-score and HbA1c, with higher BMI values occurring at intermediate HbA1c levels. This relationship varied by age, sex, and diabetes duration, while severe hypoglycaemia and insulin pump therapy were associated with small increases in BMI z-score [31].

Birkebaek et al. reported that low HbA1c, long diabetes duration, higher insulin dose, pump treatment and experiencing a severe hypoglycemia predicted higher BMI-SDS in a multicentric cohort study [32]. At the same time, in a retrospective cohort of children with T1DM followed for at least five years, BMI-SDS, HbA1c, and daily insulin dose increased progressively over time, with a higher proportion of girls exhibiting poor metabolic control. No significant association was observed between BMI-SDS and HbA1c, suggesting that changes in body weight were not directly related to glycemic control in this cohort [33].

National cohorts similarly demonstrate that BMI-SDS increases with both age and diabetes duration, particularly during puberty. Children diagnosed at older ages and those entering puberty after diagnosis exhibit the steepest BMI-SDS increases. Puberty represents a critical period, during which physiological IR necessitates higher insulin doses, potentially amplifying anabolic effects and promoting fat accumulation [21,34].

Sex-specific differences are consistently observed. Girls with T1DM tend to have higher BMI-SDS and a greater likelihood of belonging to unfavorable weight-gain trajectories compared with boys [34,35].

Treatment-related factors also contribute to post-diagnostic weight gain. Higher total daily insulin doses and intensive insulin therapy have been associated with increased BMI and central adiposity, reflecting the anabolic properties of insulin and compensatory eating to prevent hypoglycemia. While advanced technologies such as hybrid closed-loop systems improve glycemic control, available evidence suggests they do not fully prevent excess weight gain [36,37].

Sociodemographic factors also play a role in post-diagnostic weight trajectories in youth with T1DM. Lower socioeconomic status, minority ethnicity, and family-related factors have been consistently associated with higher BMI and less favorable adiposity trajectories in children and adolescents with T1DM, as demonstrated in large longitudinal cohort analyses. In particular, a longitudinal study from the SEARCH for Diabetes in Youth cohort showed that youth from socioeconomically disadvantaged backgrounds and certain racial/ethnic minority groups were more likely to follow persistently high or increasing BMI trajectories over time [38].

Behavioral factors further modulate post-diagnostic weight outcomes. Physical inactivity, dietary patterns, and disordered eating behaviors have all been linked to excess weight gain and poorer metabolic profiles in adolescents with T1DM, with adolescents appearing particularly vulnerable [39,40,41]. Studies examining eating behaviors in youth with T1DM report higher rates of maladaptive eating patterns among those with overweight or obesity, which may contribute to sustained weight gain and glycemic instability [26,42,43,44,45,46,47].

Overall, the findings indicate that overweight and obesity are highly prevalent and often progressive after T1DM diagnosis, driven by interactions between pubertal physiology, sex, treatment intensity, and broader obesogenic environments. These post-diagnostic weight trajectories have important implications, as excess adiposity in youth with T1DM is closely linked to IR and early cardiometabolic risk.

### 3.5. Double Diabetes Phenotype: Evidence and Prevalence

To clarify the evidence supporting the emergence of the dual phenotype of obesity and T1DM in pediatric populations, we summarized in Table 3 key studies reporting excess adiposity, IR, post-diagnostic weight trajectories, cardiometabolic complications, and treatment approaches in children and adolescents with T1DM.

Large registry-based studies demonstrate that a substantial proportion of children and adolescents with T1DM meet criteria consistent with a double diabetes phenotype. In the T1DM Exchange registry including 11,348 youth aged 2–18 years, 22% were overweight and 14% were obese, and obese participants had significantly higher odds of hypertension and dyslipidemia compared with their normal-weight peers [21]. These cardiometabolic abnormalities mirror those typically observed in youth with T2DM, supporting the concept of phenotypic overlap.

Similarly, European registry data (SWEET and national cohorts) report that 30–40% of youth with T1DM are overweight or obese, with 10–20% meeting criteria for obesity, creating a large population at risk for IR-related complications [20,50]. Case–control data further show that abdominal obesity—a hallmark of IR—is more prevalent in children with T1DM than in non-diabetic controls [30].

Several studies directly demonstrate increased IR in youth with T1DM and excess adiposity [51,52,53,54]. In autoantibody-positive relatives and individuals with early T1DM, higher BMI percentile was strongly associated with elevated HOMA-IR and reduced Matsuda index, both predictive of progression to dysglycemia and overt diabetes [27]. In established T1DM, obese youth require higher insulin doses per kilogram body weight, reflecting peripheral IR rather than absolute insulin deficiency alone [55,56].

The double diabetes phenotype is also characterized by early cardiovascular and hepatic involvement. Obese youth with T1DM have a significantly higher prevalence of hypertension, dyslipidemia, and subclinical vascular disease, including increased arterial stiffness and carotid intima–media thickness, independent of glycemic control [57,58,59].

In a cross-sectional study of 669 patients with T1DM or T2DM aged 2–19 years from North America, youth with T1DM and obesity, particularly in late adolescence, exhibited an adverse lipid profile comparable to that observed in adolescents with T2DM, suggesting that weight status, along with glycemia control, was an important risk factor that was associated with dyslipidemia [60]. Other studies comparing lipid profiles in overweight patients with T1DM and T2DM suggested had no difference in absolute LDL levels [61].

In addition, recent studies demonstrate a strong association between obesity in T1DM and metabolic-associated fatty liver disease (MAFLD), further reinforcing overlap with T2DM-related pathology [48,62].

Several studies reported obesity-related complications in youth with T1DM beyond traditional cardiometabolic outcomes. Increased body mass was linked to structural alterations of plantar soft tissues, including greater fat pad thickness and increased plantar fascia thickness, findings that appeared independent of diabetes-related factors and likely reflected increased mechanical load [63]. Bone health-related findings were also reported, as approximately one third of youth with T1DM had inadequate calcium intake, with female sex during adolescence and obesity identified as risk factors [64].

Emerging evidence suggests that alterations in the gut microbiome may contribute to both obesity and metabolic dysregulation in T1DM [65,66]. Studies in pediatric T1DM have demonstrated gut dysbiosis characterized by reduced microbial diversity, altered Firmicutes/Bacteroidetes ratios, and changes in short-chain fatty acid-producing bacteria, findings that have been linked to inflammation, impaired intestinal barrier function, and immune dysregulation. In parallel, obesity-related microbiome changes have been associated with IR, branched-chain amino acid metabolism, and chronic low-grade inflammation. Recent studies in youth with T1DM and obesity have identified distinct microbial and metabolite signatures compared with lean individuals with T1DM, suggesting that the gut microbiome may contribute to the development of the double diabetes phenotype through both metabolic and immunologic pathways. However, current evidence remains preliminary and largely observational.

In a pilot study of adolescents with T1DM, obesity was associated with differences in gut microbiome composition compared with lean peers, including significant variation in β-diversity (*p* = 0.013) and a higher Prevotella-to-Bacteroides ratio in the obese group (*p* = 0.0058). Functional analyses indicated altered microbial metabolic pathways related to amino acid metabolism, and stool short-chain fatty acid levels were higher in obese participants (*p* < 0.05) [66].

Genetic findings across the included studies indicate that while HLA class II genotypes remain the primary determinants of T1DM susceptibility, non-HLA variants associated with obesity and IR—such as loci in or near FTO and MC4R—influence the disease phenotype rather than autoimmune initiation [36,67,68]. Mendelian randomization analyses provide causal evidence for this link, estimating that a genetically predicted 1-SD increase in childhood BMI correlates with a 32% increased risk of T1DM. Crucially, the genetic burden of T2DM, quantified via genetic risk scores (T2D-GRS), has been shown to shape metabolic heterogeneity and accelerate the “tempo” of disease progression. In autoantibody-positive individuals, a higher T2D-GRS is significantly associated with a faster transition to clinical Stage 3 T1DM, likely by exacerbating the metabolic stress on remaining beta-cells [69]. This pattern supports a model where genetic predisposition to IR interacts with excess adiposity to determine the severity of the double diabetes phenotype, leading to higher insulin requirements and adverse cardiometabolic profiles post diagnosis [70,71].

## 4. Discussion

In this systematic review, we synthesized evidence from 67 studies examining obesity as a risk factor for T1DM, its influence on disease onset, and its role in shaping post-diagnostic metabolic trajectories and the emergence of a double diabetes phenotype. Collectively, the findings indicate that excess adiposity is not merely a comorbidity in T1DM, but a biologically and clinically relevant factor that may accelerate disease manifestation in susceptible individuals and substantially modify the clinical course after diagnosis.

### 4.1. Role of Obesity in the Pathogenesis of Type 1 Diabetes Mellitus

The strongest support for obesity as a risk factor for T1DM derives from large population-based cohorts and longitudinal studies in genetically at-risk individuals [13,36]. This finding extends earlier observations linking accelerated childhood growth and increased body size with rising T1DM incidence and lends strong support to the “accelerator hypothesis,” which proposes that obesity-related IR increases β-cell stress and accelerates autoimmune destruction [72].

Mechanistic evidence from autoantibody-positive relatives further reinforces this concept. The association between IR indices (HOMA-IR, Matsuda index), BMI percentile, and progression through pre-symptomatic stages of T1DM suggests that metabolic stress may act as a disease accelerator once autoimmunity is established. Importantly, these effects were observed after adjustment for insulin secretory capacity, supporting an independent role of IR rather than simply reflecting declining β-cell function [11].

At the same time, findings across pediatric cohorts at diagnosis were heterogeneous. While several studies reported a high prevalence of overweight and obesity at presentation and, in some cases, slightly younger age at diagnosis among heavier children, others did not find a consistent association between BMI and age of onset [1,17,60]. These discrepancies likely reflect differences in study design, age distribution, pubertal status, and genetic background, underscoring that obesity is neither necessary nor sufficient for T1DM development but may act as a modifier in genetically predisposed individuals.

Furthermore, in a 6-year retrospective cohort of 119 children and adolescents with T1DM, partial remission occurred in 63% of patients and was observed in nearly all overweight and obese participants. BMI Z-score trajectories differed according to remission duration, with lower initial BMI in non-remitters and convergence of BMI Z-scores over time, while daily insulin requirements did not differ between groups after 6 years [73]. Similarly, Pyziak-Skupien et al. reported that the maintenance of proper body weight in children with a newly diagnosed T1D was be the most important factor determining the incidence of clinical partial remission [74].

Genetic evidence suggests that while HLA class II genotypes drive T1DM susceptibility, non-HLA variants linked to obesity and IR (e.g., FTO, MC4R) primarily influence disease phenotype. Mendelian randomization studies indicate that higher genetically predicted BMI increases T1DM risk, and higher T2D genetic risk scores are associated with faster progression to clinical disease. Together, these findings support a model in which genetic predisposition to IR interacts with adiposity to shape the double diabetes phenotype [69,70,71].

### 4.2. Determinants of Post-Diagnostic Weight Gain in Type 1 Diabetes Mellitus

A consistent and clinically important finding across registries and longitudinal cohorts is the high prevalence and progressive nature of overweight and obesity after T1DM diagnosis. Approximately one-third of youth with T1DM are overweight or obese, with prevalence estimates comparable to or exceeding those of the general pediatric population. Longitudinal trajectory analyses demonstrate that weight gain is not uniform but clusters into identifiable high-risk patterns, particularly among girls, adolescents, and those diagnosed around puberty [34]. Pubertal physiology appears to play a central role in these trajectories. Physiological IR during puberty necessitates higher insulin doses, which may amplify insulin’s anabolic effects and promote fat accumulation.

Treatment-related factors, including intensive insulin therapy and defensive eating to prevent hypoglycemia, further contribute to positive energy balance. Importantly, while diabetes technologies improve glycemic outcomes, current evidence suggests they do not fully mitigate excess weight gain, highlighting the need for complementary lifestyle and behavioral interventions [37].

Sociodemographic and behavioral factors further modulate post-diagnostic weight outcomes. Lower socioeconomic status, minority ethnicity, physical inactivity, suboptimal dietary patterns, and disordered eating behaviors were consistently associated with less favorable adiposity trajectories. These findings emphasize that post-diagnostic obesity in T1DM arises from complex interactions between biological vulnerability, treatment demands, and broader environmental determinants [38].

### 4.3. The Emergence of Double Diabetes in Youth with Type 1 Diabetes Mellitus

The convergence of autoimmune diabetes with obesity-related IR gives rise to the increasingly recognized double diabetes phenotype. Registry data demonstrate that overweight and obese youth with T1DM have markedly higher prevalence of hypertension, dyslipidemia, central adiposity, hepatic steatosis, and early vascular changes—features traditionally associated with T2DM [20]. The requirement for higher insulin doses per kilogram body weight in obese youth further supports the presence of clinically meaningful IR [51,53].

These findings have important clinical implications. The coexistence of T1DM and IR may amplify long-term cardiovascular risk and complicate glycemic management, challenging the traditional dichotomous classification of diabetes types [75]. Early identification of high-risk weight trajectories and targeted interventions addressing adiposity and insulin sensitivity may therefore be critical components of comprehensive T1DM care.

### 4.4. Obesity, the Accelerator Hypothesis, and Familial Influences

The findings of this review should also be interpreted in the context of the “accelerator hypothesis”, originally proposed by Wilkin, which posits that obesity contributes to the pathogenesis of both T1DM and T2DM by increasing insulin demand, inducing hyperinsulinaemia, and promoting IR, thereby accelerating β-cell stress and autoimmune destruction [72]. Ecological data from Finland support this concept, demonstrating that rising incidence rates of T1DM have paralleled increases in childhood body weight and height [32]. Conversely, findings from other studies do not support this hypothesis in certain populations [9].

Relatedly, the “overload hypothesis” suggests that early-life overnutrition, physical stressors such as infection or inflammation, and psychological stress may sensitize β-cells to immune-mediated damage and apoptosis, potentially contributing to an earlier clinical onset of T1DM in the context of the global obesity epidemic [76]. However, evidence supporting this hypothesis remains inconsistent. Several studies included in this review did not confirm a direct role of obesity in accelerating autoimmune activity. For example, Cedillo et al. found no association between adiposity and autoantibody burden at diagnosis, and other analyses reported that higher BMI was associated with earlier age at diagnosis only among subgroups with reduced residual β-cell function, as reflected by lower C-peptide levels [71,77]. These findings suggest that obesity may act as a disease modifier rather than a universal trigger of autoimmunity, and that its impact may depend on underlying β-cell reserve.

In addition to metabolic and structural complications, several studies reported immunologic alterations in youth with T1DM and obesity. Elevated peripheral blood Th17 cell counts were observed both in children with obesity and in those with long-standing T1DM, suggesting overlapping inflammatory profiles associated with these conditions [78]. Children with T1DM were reported to have lower leptin levels than controls, with significant differences observed between newly diagnosed patients, those with long-standing poorly controlled disease, and across BMI categories [79].

Furthermore, not all children are overweight at the time of T1DM diagnosis. Weight loss due to glycosuria, dehydration, and reduced appetite is common before clinical presentation, and several studies report that only approximately 20% of children are overweight or obese at diagnosis [20,28]. Body weight at presentation is influenced by multiple factors, including age, sex, pubertal status, degree of metabolic decompensation, glycated haemoglobin levels, and preservation of endogenous insulin secretion. These observations underscore the heterogeneity of T1DM presentation and further highlight the limitations of attributing early disease onset solely to obesity.

Importantly, familial and intergenerational factors appear to contribute substantially to obesity and metabolic risk in youth with T1DM. Adolescents’ BMI is strongly associated with parental BMI, suggesting shared genetic and environmental influences [38]. In addition, maternal IR has been linked to later age of T1DM onset in offspring, indicating that intrauterine and familial metabolic factors may modulate disease timing [72,80]. Together, these observations emphasize that obesity in T1DM should be considered within a broader familial and developmental context, supporting the need for family-centered approaches to risk assessment and intervention.

### 4.5. Genetic Considerations

Genetic findings across the included studies indicate that while HLA class II genotypes remain the primary determinants of T1DM susceptibility, non-HLA variants associated with obesity and IR influence disease phenotype rather than autoimmune initiation. These variants were linked to higher adiposity, increased insulin requirements, and adverse cardiometabolic profiles but showed no consistent association with autoantibody burden at diagnosis [67,68,71]. This pattern supports a model in which genetic predisposition to IR interacts with excess adiposity to shape disease expression and metabolic risk after onset.

### 4.6. Management of Obesity in Patients with Type 1 Diabetes Mellitus

The treatment of obesity in individuals with T1DM presents distinct therapeutic challenges compared with obesity management in the general population or in T2DM [81]. 

Nutrition is the cornerstone of obesity management in T1DM and must be carefully aligned with insulin therapy to prevent hypoglycemia. High-quality dietary patterns, structured carbohydrate counting, and avoidance of hypoglycemia-related overeating are essential to limit excess weight gain. Regular physical activity further supports insulin sensitivity, although its implementation requires individualized adjustments to minimize hypoglycemia risk [82,83,84].

Optimization of insulin therapy is a key component of weight management. Real-world data from pediatric cohorts using automated insulin delivery systems are supporting the concept of “insulin stewardship,” aiming to minimize unnecessary insulin exposure while maintaining glycemic targets [84,85,86].

Adjunct pharmacotherapy may be considered in selected patients, particularly those with obesity and IR. Metformin is the most extensively studied agent and has been associated with modest reductions in body weight and insulin requirements, although its effect on glycemic control is limited and often transient [49,85]. Glucagon-like peptide-1 receptor agonists (GLP-1 RAs) have shown promising results, with consistent reductions in body weight, insulin dose, and modest improvements in glycemic parameters [86]. Sodium–glucose cotransporter-2 (SGLT2) inhibitors may also reduce body weight and insulin dose, but their use is limited by the risk of euglycemic diabetic ketoacidosis, requiring careful patient selection and monitoring [87]. Evidence in pediatric populations remains scarce, although small studies suggest potential benefits [88,89]. Other agents, such as pramlintide, may contribute to modest weight reduction but are limited by tolerability and treatment burden [90,91].

Bariatric surgery represents a potential option for severe, refractory obesity, primarily in adults with T1DM. Available evidence suggests substantial weight loss and reduced insulin requirements, although improvements in glycemic control are variable and do not reflect remission of β-cell failure [92,93].

### 4.7. Strengths and Limitations

This review benefits from the inclusion of diverse study designs, large registry datasets, and mechanistic studies in at-risk populations. However, the overall moderate risk of bias—driven primarily by observational designs, residual confounding, and incomplete follow-up reporting—limits causal inference. Heterogeneity in obesity definitions, age ranges, and outcome measures further complicates quantitative synthesis. A key limitation of this review is the restriction to studies conducted in Europe and North America, focusing on populations of predominantly European ancestry to reduce genetic heterogeneity. While this improves comparability across studies, it limits the generalizability of the findings to more diverse populations with different genetic and socioeconomic backgrounds. Future studies employing standardized phenotyping, genetic risk stratification, and long-term prospective follow-up are needed to clarify causal pathways and inform prevention strategies.

## 5. Conclusions

In summary, the evidence indicates that obesity is an important modifier of T1DM risk and progression. Excess adiposity and IR appear to accelerate disease manifestation and contribute to adverse metabolic trajectories and the development of a double diabetes phenotype after diagnosis. These findings highlight the need to integrate weight management and cardiometabolic risk reduction into the routine care of children and adolescents with T1DM, alongside traditional glycemic targets.

## Figures and Tables

**Table 1:** Prevalence of Excess Weight in International Pediatric T1DM Registries.

Registry/Study	Region	Overweight Prevalence (%)	Obesity Prevalence (%)	Combined Prevalence (%)
SWEET Registry (n = 23,026)	International	22.3 (M)/27.2 (F)	7.3 (M)/6.8 (F)	31.8% (Overall)
T1DM Exchange (n = 11,348)	USA	22.0%	14.0%	36.0%
German/Austrian Registry	Europe	12.5%	2.8%	15.3%
Nordic Countries Cohort	Scandinavia	18.6%	18.5%	37.1%

**Table 2:** BMI Status Trajectory over 5 Years Following T1DM Diagnosis.

Years After Diagnosis	Normal Weight (%)	Overweight (%)	Obese (%)	Combined Overweight/Obese (%)
0 (Diagnosis)	63.2%	15.8%	19.7%	35.5%
3 Years	-	-	-	50.0%
5 Years	47.2%	-	-	52.8%

**Table 3:** Characteristics of key studies regarding the development of the dual phenotype of obesity and T1DM.

Study	Design	Population	Key Findings	Relevance to Dual Phenotype
Redondo et al. [21]	Registry-based cross-sectional study	11,348 youth with T1DM (2–18 years), T1DM Exchange	22% overweight, 14% obese; obesity associated with higher odds of hypertension and dyslipidemia	Demonstrates high prevalence of excess weight and cardiometabolic risk in pediatric T1DM
Petrelli et al. [11]	Prospective cohort (TrialNet)	Autoantibody-positive relatives at risk of T1DM	Higher BMI associated with increased IR; higher HOMA-IR predicted progression to clinical T1DM	Suggests obesity-related IR may accelerate disease progression
Phelan et al. [28]	Longitudinal international cohort	11,513 youth with T1DM	Distinct BMI trajectories, including persistently high and increasing BMI patterns	Identifies high-risk subgroups for obesity development
De Keukelaere et al. [27]	Longitudinal cohort	390 children with T1DM followed from diagnosis	BMI-SDS increased with age and diabetes duration, especially in girls and during puberty	Supports role of sex, puberty, and disease duration in obesity development
Kącka-Stańczak et al. [48]	Observational clinical study	Children/adolescents with T1DM	Obesity associated with IR, MAFLD, and vascular alterations	Demonstrates overlap with T2DM-like metabolic complications
Gonzalez et al. [49]	Retrospective cohort	24 youth (10–20 years) with T1DM and obesity treated with GLP-1RA	Significant reductions in weight, BMI, and insulin dose; improvement in glycemic parameters	Suggests reversibility of IR component

## Data Availability

This systematic review did not generate new primary data.

## Abbreviations

The following abbreviations are used in this manuscript:

T1DM	Type 1 diabetes mellitus
T2DM	Type 2 diabetes mellitus
BMI	Body mass index
CVD	Cardiovascular disease
MAFLD	Metabolic-associated fatty liver disease
IR	Insulin resistance
